# MiR-101-3p and miR-106b-5p roles in EMT pathway: prognostic and therapeutic insights for luminal breast cancer

**DOI:** 10.1186/s43046-025-00304-z

**Published:** 2025-07-21

**Authors:** Gehad Tarek, Manar S. Fouda, Mohamed M. Omran, Gehan Safwat, Mahmoud M. Kamel, Abdel Hady A. Abdel Wahab

**Affiliations:** 1 October University for Modern Sciences and Arts (MSA), Giza, Egypt; 2 Helwan University, Cairo, Egypt; 3 National Cancer Institute, Cairo University, Cairo, Egypt; 4 Baheya Foundation for Early Detection & Treatment of Breast Cancer, Giza, Egypt

**Keywords:** Breast cancer, Noncoding RNA, EMT, MicroRNA, Luminal subtype

## Abstract

**Introduction:**

Breast cancer is considered to be the most common cancer that affects women worldwide, where it accounts for approximately 38.8% of all cancer cases among females. Luminal subtypes are the most prevalent in Egypt. Small noncoding RNAs also called microRNAs (miRNAs) influence gene expression posttranscriptionally. Since they regulate the epithelial-mesenchymal transition process, which is vital for tumor invasion and metastasis, microRNAs play a critical role in the progression of cancer.

**Methods:**

This study has investigated the expression profiles of four microRNAs (miR-101-3p, miR-106a-5p, miR-106b-5p, and miR-130b-5p) and their impacts on genes associated with epithelial-mesenchymal transition (EMT) in luminal breast cancer. Tissue samples from 43 luminal breast cancer patients and 18 controls have been studied via real-time PCR (RT-qPCR). The association between the expression levels was evaluated using the Pearson correlation test. The correlation between the measured variables and numerous clinicopathological characteristics was assessed using the linear regression test.

**Results:**

The results demonstrated that miR-101-3p, miR-106a-5p, and miR-106b-5p were significantly dysregulated, highlighting their possible role as oncogenes or tumor suppressors in the development of breast cancer. EMT markers, especially Twist, SNAI1, and E-cadherin, show significant alterations, indicating the activation of EMT pathways in luminal breast cancer. Correlation analysis showed interactions between miRNAs and EMT-related genes, showing a negative correlation between miR-101-3p and SNAI1, as well as a positive correlation between Twist and miR-106a-5p. Moreover, logistic regression analysis associated expression levels of those miRNAs with clinicopathological characteristics, such as body weight, age, and tumor laterality.

**Conclusion:**

These findings highlight the leading role of miR-101-3p and miR-106b-5p in the progression of luminal breast cancer via interacting with the EMT process and their potential as diagnostic, prognostic, and therapeutic targets.

## Background

Breast cancer is the predominant malignancy impacting women globally, and Egypt is no exception, accounting for around 38.8% of all cancer cases among women [1]. The disease exhibits heterogeneity, with multiple molecular subgroups categorized based on hormone receptor expression and HER2 (human epidermal growth factor receptor 2) status. The primary subtypes include luminal A, luminal B, HER2-enriched, and triple-negative breast cancer (TNBC) [2]. Luminal breast cancers (luminal A and luminal B) are distinguished by the presence of estrogen receptors (ER) and represent the predominant subtypes in Egypt [3].

Luminal breast cancer is characterized by its ability to become more aggressive and metastasize to other organs [4]. This occurs through epithelial-to-mesenchymal (EMT), a physiological mechanism in which carcinoma cells lose their epithelial traits and acquire motility to invade surrounding tissues [5]. In breast cancer, this process is mediated by various dysregulated signaling pathways contributing to disease progression and chemotherapy resistance [6].

Regardless of the existence of targeted medicines, luminal breast cancer continues to pose treatment challenges owing to its propensity for late recurrence, often occurring years after the first intervention [7]. Conventional endocrine therapy, although successful in the short term, frequently does not avert long-term recurrence, especially in the luminal B subtype. The elevated recurrence rates are mostly ascribed to resistance mechanisms that undermine the long-term effectiveness of these therapies [8]. This highlights the urgent need for novel therapeutic strategies that can enhance treatment effectiveness and reduce recurrence rates.

A potential research area is microRNAs (miRNAs), which are tiny noncoding RNAs that modulate gene expression posttranscriptionally. MicroRNAs are pivotal in breast cancer progression, especially by regulating epithelial-mesenchymal transition, which is fundamental to tumor invasion and metastasis [9]. According to their context, miRNAs can either facilitate or inhibit EMT, establishing them as crucial regulators in luminal breast cancer [10]. Specific miRNAs inhibit EMT via slug dependent mechanisms [11]. In contrast, certain miRNAs may facilitate EMT, hence boosting the metastatic capacity of breast cancer cells [12].

miR-101-3p acts as a tumor suppressor, and its downregulation facilitates metastasis by upregulating COX-2 and MMP1 [13]. In contrast, miR-106-a-5p and miR-106b-5p function as oncogenes, promoting cell proliferation and survival by targeting tumor suppressors like PTEN and enhancing migration and invasion through genes such as FUT6 and CNN1 [14, 15]. miR-130b-5p also exhibits oncogenic behavior by activating the PI3 K/Akt pathway via PTEN suppression and promoting stromal remodeling through exosome-mediated downregulation of SPIN90, contributing to an aggressive tumor microenvironment [16].

To identify novel biomarkers as well as therapeutic targets for luminal breast cancer, it is necessary to investigate how specific miRNAs regulate the cell cycle and EMT genes in this subtype. The primary aim of this study is to clarify the significance of the selected panel of miRNAs in luminal breast cancer and to elucidate their pivotal roles in modulating the EMT pathway.

## Methods and materials

### Specimen collection

Fresh tissue samples were obtained from 43 luminal breast cancer patients diagnosed at Baheya Hospital between February 2022 and December 2023 using core needle biopsy technique. The tissues of 18 patients were also collected as controls. All of the tissues were collected prior to performing any kind of treatment. A portion of the tissue was preserved in buffered formalin for the histological examination, while the other piece was simultaneously stored at − 80 °C for additional investigation. This study was approved by Egypt’s Baheya Research Ethical Committee (IRB no. 00012829), the Baheya Foundation for Early Detection & Treatment of Breast Cancer, Egypt. In compliance with the Declaration of Helsinki, the norm of ethics established by the World Medical Association, each participant provided their informed permission. The various clinicopathological characteristics collected from all participants are presented in Table 1.

**Table 1:** Clinical characteristics of luminal breast cancer patients

Variables	*N* (%)
Clinical characteristics	
Age at diagnosis (years), mean ± SD	59 ± 12.8
Menopausal status	
Premenopausal	36 (84%)
Postmenopausal	7 (16%)
Family history	
Present	9 (21%)
Absent	34 (79%)
BMI, mean ± SD	34.07 ± 6.62
BMI category	
Normal weight	5 (12%)
Overweight	14 (32%)
Obese	24 (56%)
Pathological characteristics	
Affected breast	
Right	25 (58%)
Left	18 (42%)
Molecular subtype	
A	40 (93%)
B	3 (7%)
HER2-enriched	0 (0%)
TNB	0 (0%)
Histological type	
Ductal carcinoma	40 (93%)
Lobular carcinoma	3 (7%)
Pathological grade	
Grades 0–1	6 (14%)
Grades 2–3	37 (86%)
Tumor size (cm)	
< 5	34 (79%)
≥ 5	9 (21%)
Tumor stage	
Stages I–II	23 (54%)
Stages III–IV	20 (46%)
Lymph node metastasis	
Negative	23 (35%)
Positive	28 (65%)
Distant metastasis	
Absent	43 (100%)
Present	0 (0%)

### Quantitative real-time polymerase chain reaction (RT‒qPCR)

Using each of the miRTarBase and the ENCORI/StarBase databases, an attempt was conducted to investigate a set of microRNAs associated with genes that regulate the EMT process. According to their findings, many EMT genes were directly and closely associated with each of miR-101-3p, miR-106a-5p, miR-106b-5p, and miR-130b-5p. Therefore, their expression levels were determined in tissues of patients with hormonal breast cancer as well as normal tissues for comparison. Frozen tissue samples were processed to isolate high-quality total RNA, including microRNA, using the miRNeasy® Mini Kit (Qiagen, Germany). RNA quality and quantity were determined by a NanoDrop-100 spectrophotometer (Thermo Fisher Scientific, USA). Complementary DNA (cDNA) synthesis was performed with 1 µg of total RNA using the miScript II RNA reverse transcription kit (Qiagen, Germany) following the manufacturer’s protocol. The resulting cDNA was diluted fivefold, and 1 µL of the diluted cDNA was used for subsequent PCR. The relative expression levels of miRNAs (miR-101-3p, miR-106a-5p, miR-106b-5p, and miR-130b-5p) and EMT markers (E-cadherin, N-cadherin, vimentin, fibronectin, Twist, SNAI1, Slug, ZEB1, and ZEB2) were analyzed by PerfectStart® Green qPCR SuperMix (TransGen Biotech Co., Ltd., Beijing, China). Primer sequences were designed and obtained from Eurofins Genomics (GmbH, Germany). PCR reactions were performed on a ViiA7 real-time PCR system (Applied Biosystems, Foster City, CA, USA) using the following thermocycling protocol: an initial denaturation step at 95 °C for 15 min, followed by 40 cycles of 95 °C for 20 s and 60 °C for 60 s. Each sample was analyzed in duplicate, and relative expression levels were calculated using the 2^^−∆∆Ct^ method [17]. RNU6 served as the normalization control for miRNAs, while GAPDH was used as the endogenous reference for EMT markers.

### Statistical analysis

Data analysis was conducted using IBM SPSS Statistics v26.0 software (SPSS Inc., Chicago, Ull, USA). Median ± IQR or mean ± SD were used. The Mann–Whitney test was used to compare those in two groups. To evaluate the link between two distinct parameters that were measured, Pearson correlation analysis was used. The association between miRNA levels and several clinicopathological characteristics of patients with breast cancer was assessed using logistic regression. *P*-values less than 0.05 were regarded as statistically significant differences.

## Results

### Patient’ characteristics

Forty-three patients diagnosed with breast cancer were included in this study; the average age at diagnosis was close to 59 years, and 84% of them were premenopausal. Their family history was not significant (79%). Only about 12% of the patients were of normal weight, indicating the majority were overweight or obese. Only 7% of patients were subtype B breast cancer, whereas 93% of patients were subtype A breast cancer, with the right side of the breast being more severely affected than the left. Furthermore, ductal carcinoma was more common than lobular carcinoma, and 86% of patients had a late tumor grade, whether it was 2 or 3. The most common tumor size in this study was less than 5 cm, and the highest percentage of patients (65%) had tumor spread to lymph nodes in the absence of any metastatic patients as described in Table 1.

### MicroRNA expression levels in patients with luminal breast cancer

As described in the methodology chapter, a group of four microRNAs, including miR-101-3p, miR-106a-5p, miR-106b-5p, and miR-130b-5p, were selected from a variety of available databases to determine their correlation and significance in controlling the EMT process in the cell. The levels of these microRNAs were assessed in tissues from luminal breast cancer patients compared with those of normal controls. The findings indicated that in comparison to the control, the level of miR-101-3p was significantly reduced by 37.3-folds (*p* = 0.0001). The data also showed an unexpected 9.5-fold reduction in miR-106a-5p levels (*p* = 0.023) compared to the control. Nevertheless, the results showed that the amount of miR-106b-5p had increased significantly, up to 5.13-folds above the average for the control group (*p* = 0.042). However, the study additionally found that there were no appreciable changes in the levels of miR-130b-5p (*FC* = 1.23, *p* = 0.091).

**Fig. 1:**
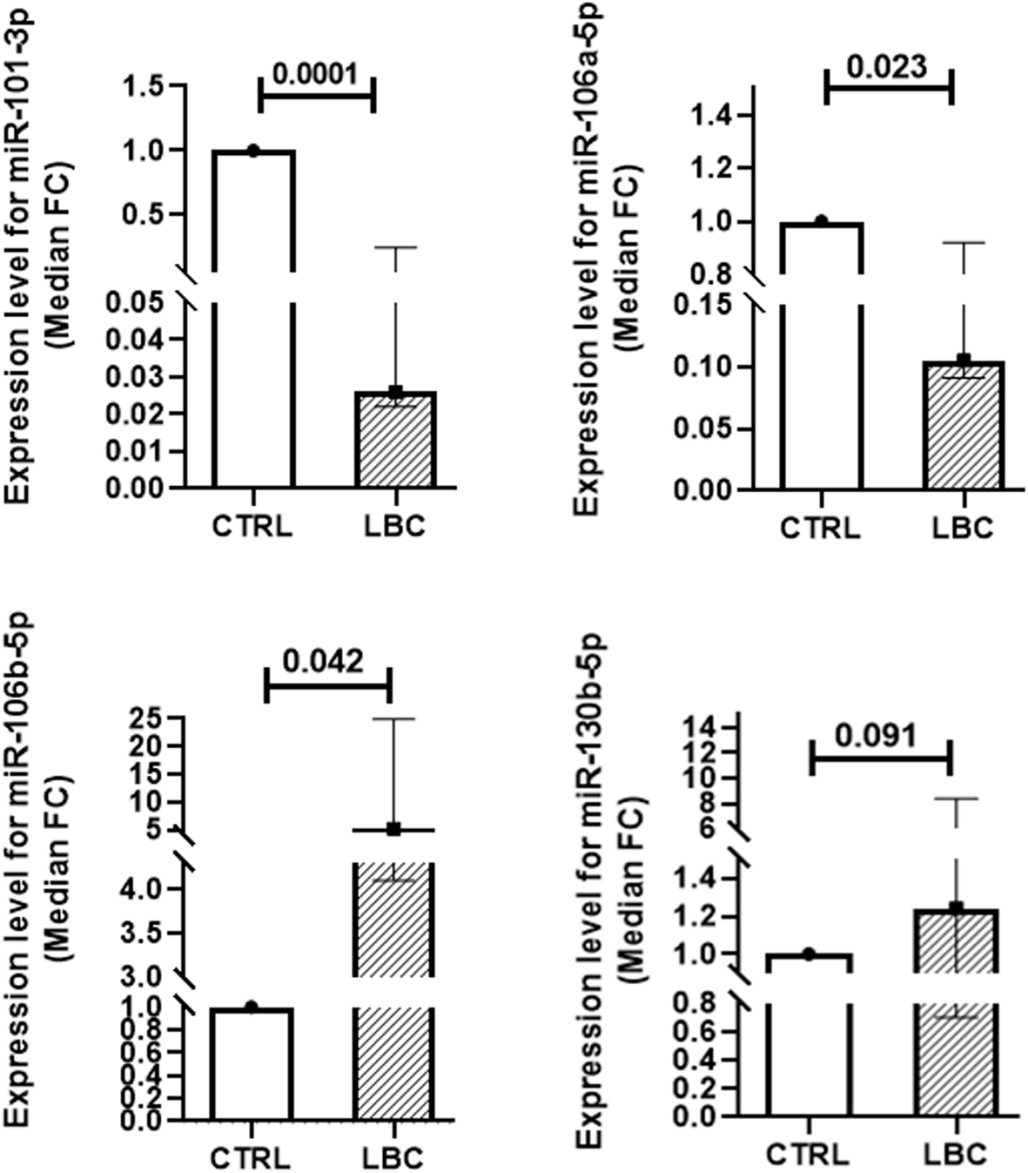
Expression levels (median fold change) of different miRNAs including miRNA-101a-3p, miRNA-106a-5p, miRNA-106b-5p, and miRNA-130b-5p in luminal breast cancer tissues (LBC) compared to noncancerous tissues (CTRL). Statistically significant results were defined by *p*-values < 0.05

According to the data above, miR-106b-5p is thought to have an oncogenic role in breast cancer, whereas miR-101-3p and miR-106a-5p both function as onco-suppressors. The results for each miRNA studied are presented in Fig. 1.

### Deregulation of EMT gene expression in luminal breast cancer patients

**Fig. 2:**
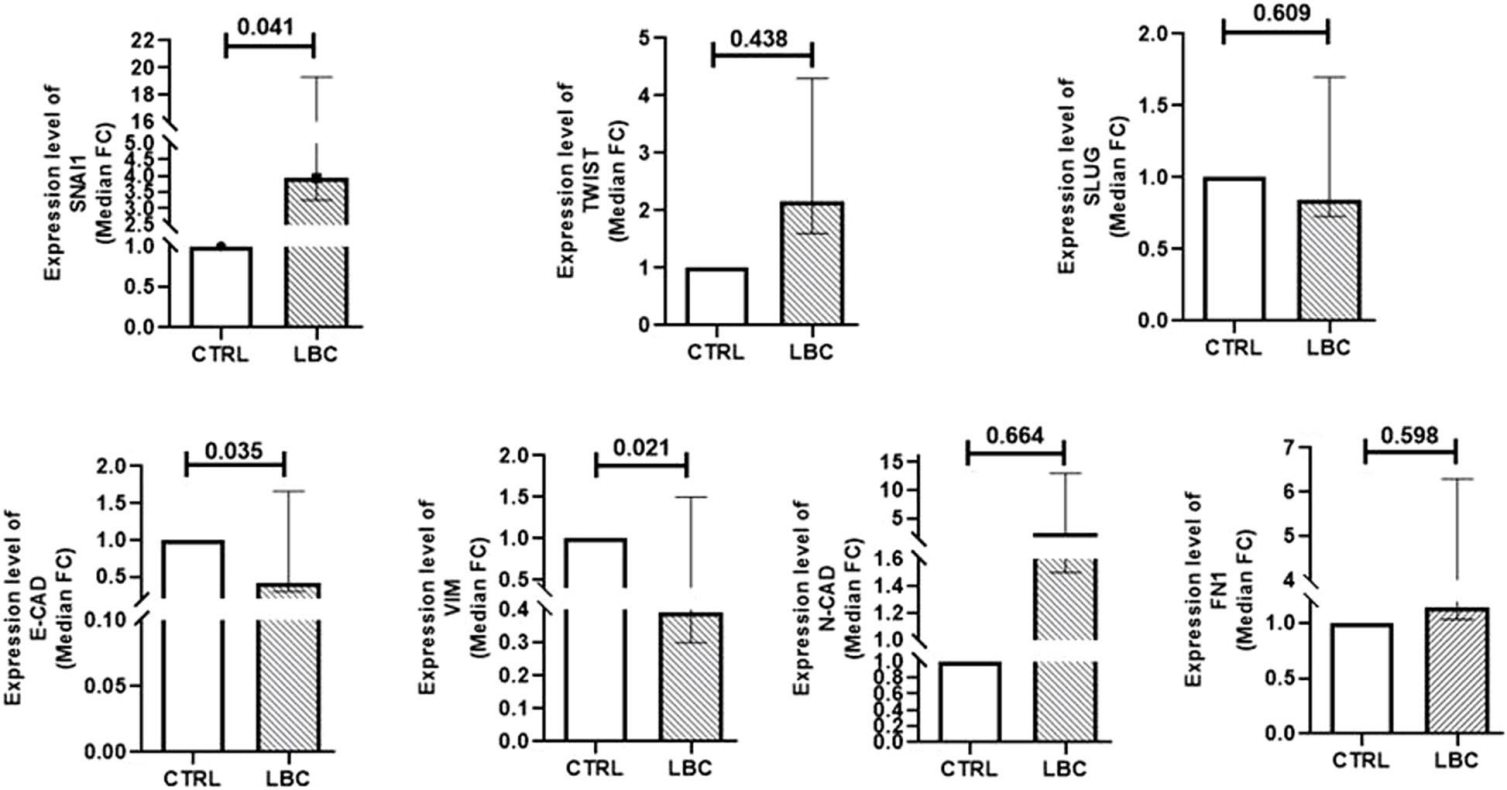
Expression levels (median fold change) of different EMT-associated genes including SNAI 1, TWIST, SLUG, E-CAD, VIM, N-CAD, and FN1 in luminal breast cancer tissues (LBC) compared to noncancerous tissues (CTRL). Statistically significant results were defined by *p*-values < 0.05

The rate of expression of a group of genes involved in the EMT process was investigated in this study. The present data showed a significant decrease in the level of E-cadherin, reaching a decrease of more than half (*FC* = 0.411, *p* = 0.035). There is also an unexpected decrease in the level of the vimentin gene (*FC* = 0.39, *p* = 0.02). Both the Twist and SNAI1 levels were elevated. The SNAI1 level increased significantly by 3.94 times (*p* = 0.041), but as noticed in the Twist level (*FC* = 2.15, *p* = 0.438), this increase did not reach significance. An increase was observed in the level of the N-cadherin gene, but it did not reach the significant value (*FC* = 2.354, *p* = 0.664). Our data did not indicate any significant alteration in the level of Slug (*FC* = 0.842, *p* = 0.609). At the same time, no significant changes were observed in the fibronectin level (*FC* = 1.136, *p* = 0.598), as is indicated in Fig. 2.

### Correlation between EMT markers and miRNAs

**Fig. 3:**
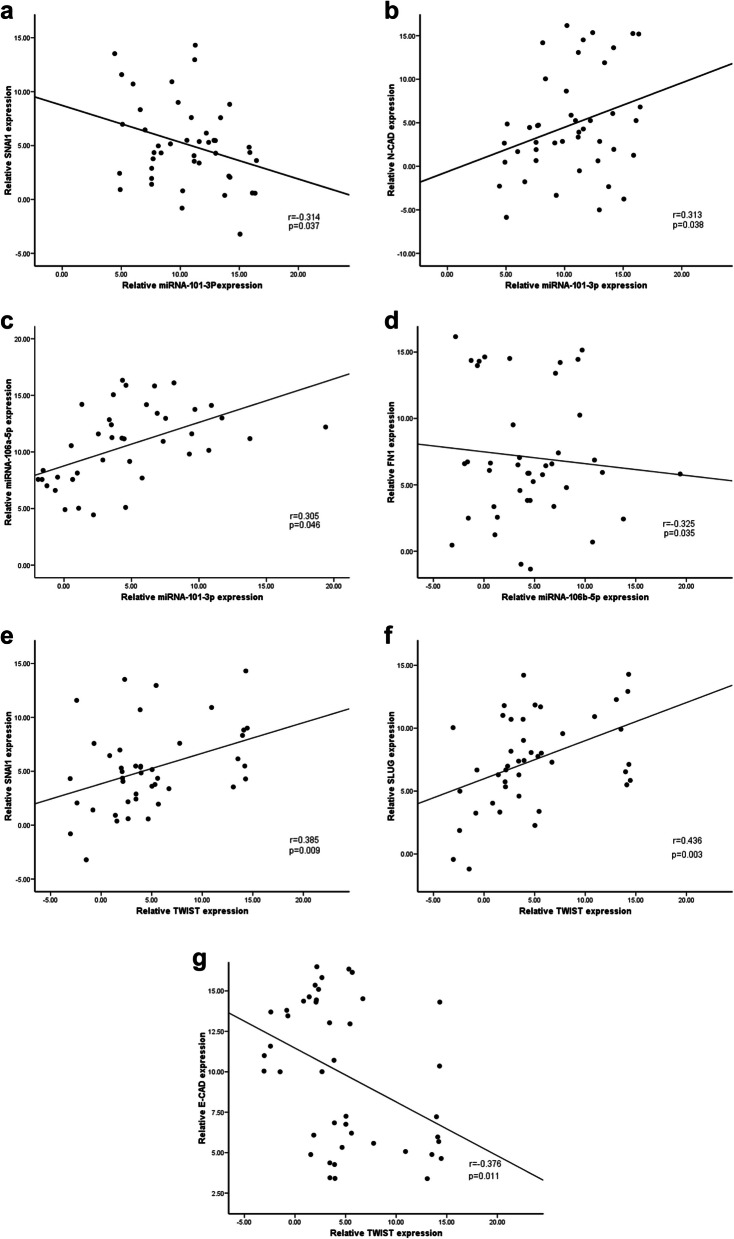
Correlation analysis between miRNA expression levels and EMT-associated genes (**a**, **b**, **c**, **d**, **e**, **f**, **g**). Pearson correlation coefficients (*r*) and corresponding *p*-values are shown for each comparison. Statistically significant results were defined by *p*-values < 0.05

The study identifies significant correlations for a subset of miRNAs (miR-101-3p, miR-106a-5p, miR-106b-5p, miR-130b-5p) and EMT markers (e.g., SNAI1, Twist, Slug, E-cadherin, N-cadherin). The research results demonstrated a strong and substantial inverse correlation between the levels of SNAI1 and miR-101-3p (*r* = − 0.314, *p* = 0.037). Additionally, an inverse correlation between the levels of fibronectin and miR-106b-5p was identified, and this association reached the significant value (*r* = − 0.325, *p* = 0.035). Furthermore, there is a significant direct correlation between the miR-101-3p level and each of N-cadherin (*r* = 0.313, *p* = 0.038) and miR106a-5p level (*r* = 0.539, *p* = 0.0002). Twist level exhibited a significant direct correlation with miR-106a-5p (*r* = 0.305, *p* = 0.046). miR-130b-5p did not show any significant correlation with each of the EMT markers examined in the current study. This test also demonstrated a strong and direct significant correlation between each twist level with SNAI1 (*r* = 0.385, *p* = 0.0097) and Slug (*r* = 0.436, *p* = 0.0030), whereas the relationship between Twist level and E-cadherin was inverse, with an increase in Twist accompanied by a significant reduction in E-cadherin levels (*r* = − 0.376, *p* = 0.011). Some of these correlations are illustrated in Fig. 3a, b, c, d, e, f, and g.

### Correlation between the investigated miRNAs and various clinicopathological features of patients with breast cancer

MicroRNAs have previously been identified as prognostic factors for cancer patients. Through the investigation of the four microRNAs and their associations to various clinical and pathological features of patients, an attempt was made to understand this correlation using logistic regression analysis in the current study. Since the level of miR-101-3p was shown to decrease as body weight increased, the study’s findings demonstrated an important association between the patients’ body weight and miR-101-3p level as shown in Table 2. This suggests a potential regulatory interaction that may influence key intracellular processes. As observed through some of these various correlations in Table 3, the study also found a significant correlation between patient age and the level of miR-106a-5p, with lower levels observed in premenopausal women. This highlights the significance of age in changes in the level of miR-106a-5p inside the cell. Lastly, tumor laterality was found to be associated with miR-106b-5p levels, and patient with tumor on the right side of the breast exhibits significantly higher expression compared to those with tumors on the left (Table 4). In contrast, no significant correlation was found between miR-130b-5p level and each of the clinical and pathological features evaluated (Table 5).

**Table 2:** Logistic regression analysis in miR-101a-3p expression level with odds ratio and 95% CI for various clinicopathological variables in luminal breast cancer

Variables	*β*	*SE*	*OR*	*p*-value	95% *CI* lower	95% *CI* upper
Age at diagnosis	− 0.544	1.230	0.581	0.661	0.051	6.570
Family history	− 1.520	1.084	0.219	0.161	0.026	1.829
BMI	− 2.169	1.131	0.114	0.050	0.012	1.048
Affected breast	− 0.736	1.200	0.479	0.540	0.046	5.031
Pathological grade	− 0.847	1.272	0.429	0.505	0.035	5.189
Tumor size	− 0.602	1.203	0.548	0.617	0.052	5.792
Lymph node status	0.519	1.202	1.680	0.666	0.159	17.719
Tumor stage	0.580	1.054	1.789	0.582	0.226	14.099

*β* regression coefficient, *SE* standard error, *OR* odds ratio, *CI* confidence interval, *BMI* body mass index

**Table 3:** Logistic regression analysis in miR-106a-5p expression level with odds ratio and 95% CI for various clinicopathological variables in luminal breast cancer

Variables	*β*	*SE*	*OR*	*p*-value	95% *CI* lower	95% *CI* upper
Age at diagnosis	0.276	0.116	2.600	0.017	1.074	1.690
Family history	0.956	0.783	1.350	0.222	0.560	12.069
BMI	0.300	0.942	2.417	0.741	0.213	8.551
Affected breast	0.904	0.758	0.276	0.233	0.599	10.917
Pathological grade	0.651	1.001	1.917	0.516	0.270	13.631
Tumor size	0.013	0.732	1.013	0.986	0.241	4.257
Lymph node status	0.894	0.700	2.444	0.202	0.620	9.637
Tumor stage	− 0.261	0.696	0.770	0.707	0.197	3.010

*β* regression coefficient, *SE* standard error, *OR* odds ratio, *CI* confidence interval, *BMI* body mass index

**Table 4:** Logistic regression analysis in miR-106b-5p expression level with odds ratio and 95% CI for various clinicopathological variables in luminal breast cancer

Variables	*β*	*SE*	*OR*	*p*-value	95% *CI* lower	95% *CI* upper
Age at diagnosis	− 0.442	1.160	0.643	0.703	0.066	6.250
Family history	0.780	0.795	2.182	0.327	0.459	10.371
BMI	0.949	0.974	2.583	0.330	0.383	17.432
Affected breast	1.792	0.856	6.000	0.036	1.120	32.142
Pathological grade	0.811	0.885	2.250	0.360	0.397	12.751
Tumor size	1.243	1.035	3.467	0.230	0.456	26.327
Lymph node status	− 0.049	0.815	0.952	0.952	0.193	4.705
Tumor stage	− 0.651	0.792	0.522	0.411	0.111	2.642

*β* regression coefficient, *SE* standard error, *OR* odds ratio, *CI* confidence interval, *BMI* body mass index

**Table 5:** Logistic regression analysis in miR-130b-5p expression level with odds ratio and 95% CI for various clinicopathological variables in luminal breast cancer

Variables	*β*	*SE*	*OR*	*p*-value	95% *CI* lower	95% *CI* upper
Age at diagnosis	0.665	0.774	1.944	0.390	0.427	8.856
Family history	0.457	0.787	1.579	0.562	0.338	7.383
BMI	0.421	0.927	1.524	0.650	0.247	9.383
Affected breast	0.754	0.637	2.125	0.237	0.609	7.409
Pathological grade	0.211	0.982	1.235	0.830	0.180	8.459
Tumor size	− 0.095	0.660	0.909	0.885	0.249	3.313
Lymph node status	0.118	0.651	1.125	0.856	0.314	4.029
Tumor stage	0.123	0.638	1.131	0.847	0.324	3.952

*β* regression coefficient, *SE* standard error, *OR* odds ratio, *CI* confidence interval, *BMI* body mass

## Discussion

This study reveals novel insights into the significance of particular miRNAs and EMT-related genes in luminal breast cancer. The findings validate the notable dysregulation of miR-101-3p, miR-106a-5p, and miR-106b-5p, which may facilitate cancer progression via unique mechanisms. The observed downregulation of miR-101-3p and miR-106a-5p confirms their recognized functions as tumor suppressors. MiR-101-3p has been documented to modulate critical processes like apoptosis, cell proliferation, and epithelial-mesenchymal transition (EMT) by targeting oncogenic pathways such as EZH2 and COX-2, all associated with breast cancer progression [13]. Unexpectedly, miR-106a-5p was significantly downregulated in our investigation, contradicting the findings of a current study, which indicated that miR-106a-5p regulates epithelial-mesenchymal transition (EMT) and suppresses tumor cell proliferation and migration in breast cancer models. It specifically targets genes associated with the epithelial-mesenchymal transition (EMT) pathway, including E-cadherin and *β*-catenin, while also regulating apoptosis via pathways such as p53 and Bax/Bcl-2 signaling [14]. In contrast, the increase of miR-106b-5p, which facilitates tumor development and metastasis via mechanisms such as PTEN suppression and Rho/ROCK1 signaling activation, emphasizes its function as an oncogene in breast cancer [15]. Although miR-130b-5p did not exhibit significant dysregulation in this investigation, accumulating data underscores its dual involvement in cancer. In many breast cancer subtypes, miR-130b-5p has been documented to modulate cell migration, invasion, and proliferation [16].

This research demonstrates significant changes in the expression of critical EMT-related genes in luminal breast cancer tissues, offering insights into the molecular basis of cancer development. The elevated level of Twist and SNAI1, essential transcriptional regulators of EMT, along with diminished E-Cadherin levels, validates the activation of EMT pathways. The unexpected decline in vimentin levels contests the traditional EMT concept, underscoring potential subtype-specific EMT dynamics. These results align with prior research that identifies these variables as promoters of the mesenchymal phenotype and facilitators of the invasive characteristics of cancer cells [18, 19].

The current study’s results show significant insights into the correlation between EMT markers and miRs, elucidating the molecular regulatory networks that govern EMT in cancer progression. Pearson’s correlation analysis identified multiple significant associations between EMT-related genes and miRNAs, indicating that certain miRNAs may influence EMT indicators and play a role in the regulation of cellular processes. A significant negative correlation was identified between SNAI1 and miR-101-3p, indicating that elevated SNAI1 levels correspond to diminished expression of miR-101-3p. SNAI1 is a prominent transcriptional repressor that facilitates EMT by diminishing the expression of epithelial markers, including E-cadherin [20]. MiR-101-3p has been documented contributing to the inhibition of EMT in several malignancies, and our findings corroborate the hypothesis that miR-101-3p may suppress EMT via the regulation of SNAI1. The found adverse connection indicates that miR-101-3p may function as a negative regulator of SNAI1, potentially hindering the EMT process and, consequently, tumor growth. A notable positive association between miR-101-3p and N-cadherin was observed, suggesting that miR-101-3p regulates the expression of mesenchymal markers. This is very surprising, considering miR-101-3p is frequently linked to the inhibition of EMT markers [21]. This link may indicate the intricate regulatory mechanisms of miR-101-3p, which may be influenced by the cellular environment or the stage of tumor progression. A notable connection between miR-101-3p and miR-106a-5p underscores the possibility of synergistic regulation by these two miRNAs in the modulation of EMT indicators. An inverse connection between fibronectin and miR-106b-5p indicates a regulatory function of miR-106b-5p in the modulation of extracellular matrix (ECM) components associated with EMT [22]. Fibronectin is a crucial extracellular matrix protein linked to mesenchymal differentiation, and its downregulation due to miR-106b-5p may signify a decrease in mesenchymal traits, thereby impeding metastatic capability. Furthermore, the positive correlation between Twist and miR-106a-5p underscores the significance of miR-106 family members in modulating EMT [14, 22]. Twist, a transcription factor that promotes EMT, exhibited significant positive correlation with SNAI1 and Slug, both of which are essential regulators of EMT. The positive correlation between Twist and these parameters indicates that Twist may facilitate EMT by upregulating these markers [23, 24]. The inverse correlation between Twist and E-cadherin supports Twist’s documented function in diminishing epithelial traits and fostering the mesenchymal phenotype, hence augmenting cellular motility and invasiveness [25, 26].

Logistic regression analysis of clinicopathological correlations identified multiple significant associations between miR levels and other patient features, offering vital insights into the role of these molecular markers in cancer progression. A notable observation was the inverse correlation between body weight and miR-101-3p levels, indicating a possible association between metabolic alterations and miR expression. This link may have considerable consequences for recognizing how obesity or body mass index (BMI) could affect cancer biology. Obesity is linked to an elevated risk of various cancer types, including breast cancer, and alterations in miRNA expression profiles may be a mechanism contributing to this correlation. The correlation between body weight and miR-101-3p indicates that miRNA profiling may be utilized to assess or forecast the influence of obesity on cancer prognosis and treatment response [27–29]. A strong association was seen between patient age and miR-106a-5p levels. Lower levels of miR-106a-5p were specifically linked to premenopausal patients, underscoring the significance of age-related hormonal alterations in miRNA expression. Prior research has demonstrated that hormonal state, especially estrogen levels, can affect the production of numerous miRNAs, including those in the miR-106 family [30]. The identified age-related variations in miR-106a-5p expression may indicate fundamental biological mechanisms that influence the age-dependent aspects of breast cancer, such as tumor aggressiveness and treatment responsiveness. The study identified a strong correlation between tumor site (right versus left breast) and miR-106b-5p levels. Prior research indicates that the right and left breasts may have variations in cancer biology, possibly attributable to anatomical and hormonal influences [31]. The increased expression of miR-106b-5p in right-sided cancers may suggest that this miRNA is involved in regulating processes particular to this tumor location, potentially influencing variations in tumor behavior between the right and left breasts.

## Conclusion

These findings reinforce the significance of miRNAs as diagnostic and prognostic indicators in breast cancer. The differential expression of miR-101-3p and miR-106b-5p, together with their association with epithelial-mesenchymal transition pathways, offers significant insights into their therapeutic potential. Moreover, miR-130b’s restricted function in our study implies it may not serve as a principal regulator in luminal breast cancer; however, additional research under diverse situations could reveal hidden or context-dependent effects.

## Data Availability

The datasets used and/or analyzed during the current study are available from the corresponding author on reasonable request.

## Abbreviations

miRNA: MicroRNA
EMT: Epithelial-mesenchymal transition
ER: Estrogen receptor
TNBC: Triple-negative breast cancer
HER2: Human epidermal growth factor receptor 2
RT-qPCR: Reverse Transcription quantitative Polymerase Chain Reaction
cDNA: Complementary DNA
Ct: Cycle threshold
SD: Standard deviation
